# Deep learning assisted detection of glaucomatous optic neuropathy and potential designs for a generalizable model

**DOI:** 10.1371/journal.pone.0233079

**Published:** 2020-05-14

**Authors:** Yu-Chieh Ko, Shih-Yu Wey, Wei-Ta Chen, Yu-Fan Chang, Mei-Ju Chen, Shih-Hwa Chiou, Catherine Jui-Ling Liu, Chen-Yi Lee

**Affiliations:** 1 Department of Ophthalmology, Taipei Veterans General Hospital, Taipei, Taiwan; 2 Faculty of Medicine, National Yang-Ming University School of Medicine, Taipei, Taiwan; 3 Institute of Clinical Medicine, National Yang-Ming University School of Medicine, Taipei, Taiwan; 4 Department of Electronics Engineering, National Chiao-Tung University, HsinChu, Taiwan; 5 Neurological Institute, Taipei Veterans General Hospital, Taipei, Taiwan; 6 Department of Medical Research, Taipei Veterans General Hospital, Taipei, Taiwan; University of Oklahoma, UNITED STATES

## Abstract

**Purpose:**

To evaluate ways to improve the generalizability of a deep learning algorithm for identifying glaucomatous optic neuropathy (GON) using a limited number of fundus photographs, as well as the key features being used for classification.

**Methods:**

A total of 944 fundus images from Taipei Veterans General Hospital (TVGH) were retrospectively collected. Clinical and demographic characteristics, including structural and functional measurements of the images with GON, were recorded. Transfer learning based on VGGNet was used to construct a convolutional neural network (CNN) to identify GON. To avoid missing cases with advanced GON, an ensemble model was adopted in which a support vector machine classifier would make final classification based on cup-to-disc ratio if the CNN classifier had low-confidence score. The CNN classifier was first established using TVGH dataset, and then fine-tuned by combining the training images of TVGH and Drishti-GS datasets. Class activation map (CAM) was used to identify key features used for CNN classification. Performance of each classifier was determined through area under receiver operating characteristic curve (AUC) and compared with the ensemble model by diagnostic accuracy.

**Results:**

In 187 TVGH test images, the accuracy, sensitivity, and specificity of the CNN classifier were 95.0%, 95.7%, and 94.2%, respectively, and the AUC was 0.992 compared to the 92.8% accuracy rate of the ensemble model. For the Drishti-GS test images, the accuracy of the CNN, the fine-tuned CNN and ensemble model was 33.3%, 80.3%, and 80.3%, respectively. The CNN classifier did not misclassify images with moderate to severe diseases. Class-discriminative regions revealed by CAM co-localized with known characteristics of GON.

**Conclusions:**

The ensemble model or a fine-tuned CNN classifier may be potential designs to build a generalizable deep learning model for glaucoma detection when large image databases are not available.

## Introduction

Glaucoma is one of the leading causes of irreversible blindness [1]. Early diagnosis and intervention are key to prevent glaucoma blindness [2,3]; however, it is difficult to achieve early detection because of the asymptomatic nature of most types of glaucoma. Even in developed countries, the diagnostic rate of glaucoma is below 50% [4,5]. Population-based screening can identify glaucoma at an asymptomatic stage but is not feasible currently due to low cost-effectiveness [6]. Nevertheless, the combination of automated glaucoma detection with teleglaucoma may enable large-scale glaucoma screening targeted at high-risk populations [7].

Optic disc photography remains the fundamental approach in glaucoma screening [8,9]. Disc photography reviewed by experienced specialists can achieve similar diagnostic performance as that of advanced imaging technology such as optical coherence tomography (OCT) [10], and combining other modalities with fundus images did not necessarily increase the diagnostic power in teleglaucoma [7]. However, evaluation of the optic disc is subjective, and likely to encounter interobserver discordance even among glaucoma specialists [8,11]. Qualified interpretation of fundus images at limited cost through automated glaucoma detection using artificial intelligence may make fundus photography-based glaucoma screening feasible.

In the pre-deep learning era, automated glaucoma detection using fundus images was based primarily on the cup-to-disc ratio (CDR), and in some cases, defects of the retinal nerve fiber layer (RNFL) or the presence of parapapillary atrophy [12]; however, the CDR-based approach may miss important diagnostic information such as disc hemorrhage or have inadequate accuracy if the disc size is not adjusted [8,13]. The development of deep learning techniques in recent years, especially the use of convolutional neural network (CNN) and its variants for computer vision, has allowed improved medical image analysis through expanded capability to extract either low-level, coarse visual features, or high-level refined features [14]. CNN-based algorithm achieved a high diagnostic rate in differentiating the glaucomatous fundus from the healthy fundus [15–17], but its generalizability remains unclear because most studies verified the performance of the algorithm only in the images from the same dataset [15,16]. Furthermore, with the complicated diagnostic process of glaucoma involving both structural and functional assessment [18], it is difficult to build large fundus image datasets for the analysis of glaucoma. Nevertheless, the number of training images will affect the diagnostic performance and generalizability of a deep learning model [19,20]. Therefore, it is intriguing to know whether it is possible to build a CNN classifier for glaucoma detection using limited fundus images and to improve its applicability across different datasets by adding universal diagnostic characteristics, such as enlarged CDR to the model.

This study aimed to evaluate the performance of a CNN algorithm constructed with limited training images to discern glaucomatous change in images from different resources, benefits of including CDR estimation as an ensemble model, and the key features extracted for the classification in the CNN algorithm.

## Materials and methods

### Database

This study included fundus photographs in JPEG format of 465 non-glaucomatous eyes and 479 eyes with primary open angle glaucoma (POAG), which were collected retrospectively from the image database of the Department of Ophthalmology, Taipei Veterans General Hospital (TVGH). The fundus images were obtained from subjects with POAG at regular follow-up, and subjects who visited our department for ocular screening or disorders not affecting the optic disc and retina. This study followed the tenets of the Declaration of Helsinki, and was approved by the Institutional Review Board of TVGH. The written informed consent was waived because of the retrospective nature of the study and the analysis was performed using anonymized clinical data and images.

Eyes with POAG were diagnosed in the presence of glaucomatous disc changes with corresponding RNFL loss and reproducible visual field (VF) defects, and a normal open angle on gonioscopy. In all subjects with POAG, VF analysis using the 24–2 Swedish interactive threshold algorithm standard of the Humphrey Field Analyzer 750i (version 4.2, Zeiss-Humphrey Instruments, Dublin, CA, USA) and RNFL scanning using Cirrus HD-OCT (Model 4000; Carl Zeiss Meditec, Inc., Dublin, CA, USA) were performed. The normal eyes had an intraocular pressure (IOP) of < 21 mmHg, and a disc with normal appearance without RNFL defect. The fundus images were acquired using three fundus cameras, Canon CR-DGi NM fundus camera, Canon CX-1 hybrid mydriatic/non-mydriatic digital retinal camera, and Canon CR-2 PLUS AF non-mydriatic retinal camera (Canon USA, Inc., Lake Success, NY, USA) at 45° centered at the macula. All images included had a clear view of the disc. The images were reviewed by two glaucoma specialists (YK and CL) to confirm the diagnosis and define the vertical CDR. Standard anonymization was performed for all images before further analysis.

The fundus images were 3888 x 2552 pixels in size, with resolution of 120 dpi and bit depth of 24. The images were divided into a training set and test set for model training and evaluation based on stratified sampling with 763 images and 181 images at the training and test sets, respectively. The clinical information of the images is listed in Table 1. The CNN model established based on the training images from TVGH dataset was specified as TVGH-CNN model. In addition, images from the Drishti-GS Dataset [21], (http://cvit.iiit.ac.in/projects/mip/drishti-gs/mip-dataset2/Home.php), an open-access datasets containing training and test datasets of 50 and 51 images, respectively were used to evaluate the diagnostic ability of our model.

**Table 1:** Clinical and demographic characteristics of the image dataset from Taipei Veterans General Hospital.

	POAG	Control
**Number of images**	479	465
**Age (y)**	58.78±13.8	61.20±19.34
**Gender (Female, %)**	168 (35.07%)	226 (48.60%)
**Cup-to-disc ratio**	0.80±0.12	0.42±0.20
**Mean Deviation (dB)**	-6.89±6.01	
**Average RNFL thickness (μm)**	72.73±11.34	

Values are presented as mean ± SD or percentage (%). POAG, primary open angle glaucoma; RNFL, retinal nerve fiber layer.

### Proposed detection system

**Fig 1:**
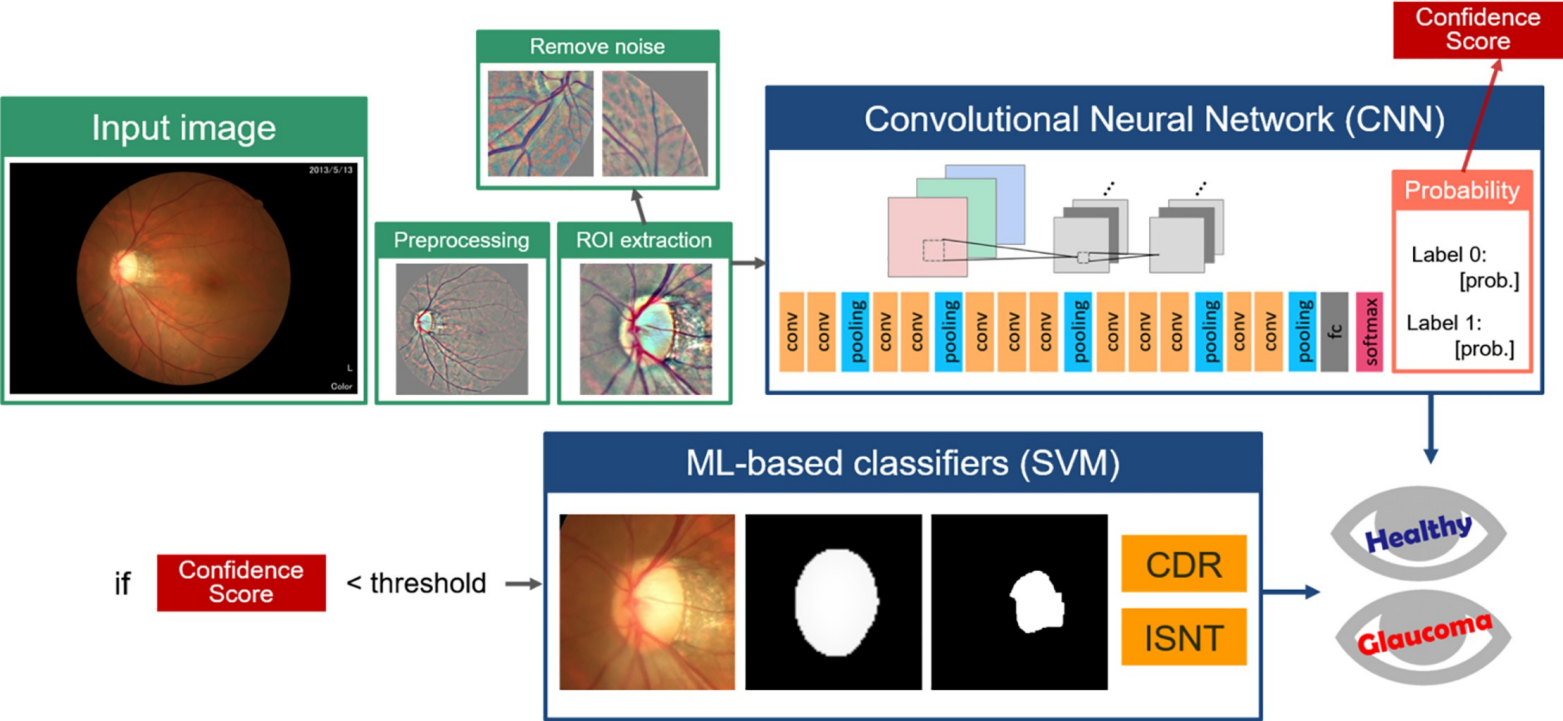
Overall Framework. Original color images were preprocessed to contrast the vessels and then submitted for ROI extraction. Image with incorrect ROI selection, referred as noise, were excluded from the CNN model. Images with low confidence score in CNN classification were forwarded to an ML-based classifier based on the key characteristics of CDR value and thinning of the superior and inferior neuroretinal rim according to the ISNT rule. CDR, cup-to- disc ratio; CNN, convolutional neural network; ML, machine-learning; ROI, region of interest; SVM, support vector machine.

The workflow of our detection system is illustrated in Fig 1. The fundus images were preprocessed and region of interest (ROI) of area of 256 × 256 pixels centered at the disc were extracted. Images with incorrectly extracted ROI were deleted automatically. Subsequently, a CNN model was used for classification. In the last layer of this CNN model, each image was classified as normal or glaucoma with a probability value, which was treated as the confidence score of the CNN classifier. If the confidence score was lower than a self-defined threshold, then the image was subjected to the support vector machine (SVM) classifier for final classification based on customized features, including enlarged vertical CDR, and thinning of the superior and inferior neuroretinal rim. The ensemble model coupling a SVM classifier based on these features was used to avoid missing subjects with moderate to advanced diseases, who will inevitable have enlarged CDR.

### Image preprocessing and ROI extraction

**Fig 2:**
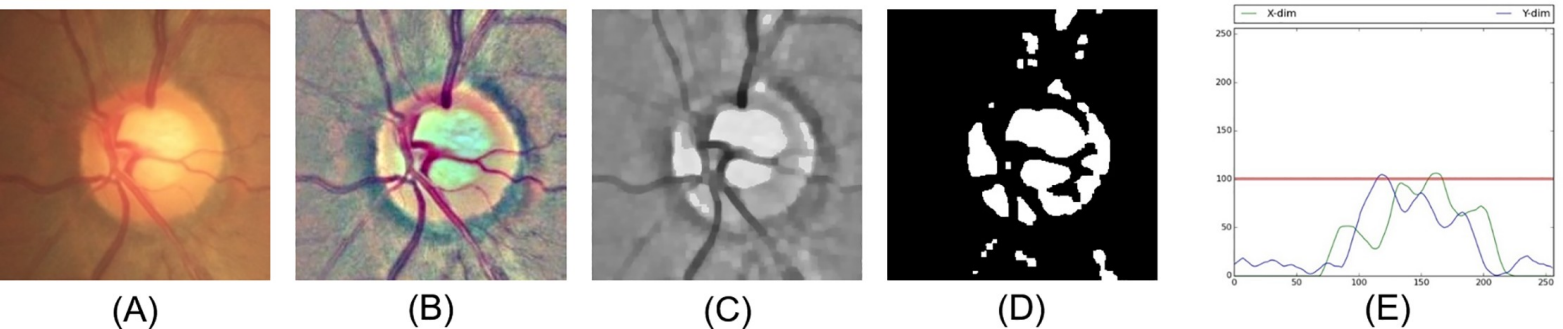
Process to determine noisy cases with incorrect ROI selection. (A) Color fundus image; (B) fundus image after preprocessing; (C) further preprocessing on the red and green channel; (D) binary display of the image; (E) histogram of the projection on x-axis and y-axis, which indicates correct ROI selection with both peaks of the axes located at the center. ROI, region of interest.

Fundus images were first preprocessed to compensate for the levels of brightness and clarity of each image. Unsharp masking by Gaussian blur method was used to enhance the optic disc structure and blood vessels. ROI detection centered at the disc was implemented to remove redundant features using the concept of guided backpropagation [22]. Following ROI extraction, images with the optic disc located at the central area of the image were considered as qualified images. Invalid cases with incorrect ROI were automatically excluded through distribution of the histogram on the red and green channel. Images with the disc centered at the ROI would show a peak at the center and were included in subsequent analysis (Fig 2). Finally, data augmentation by adding deformations and noise to the dataset was performed by rotation, shifting, sheering, and zooming using the Keras library (https://keras.io/).

### Detection based on CNN

**Fig 3:**
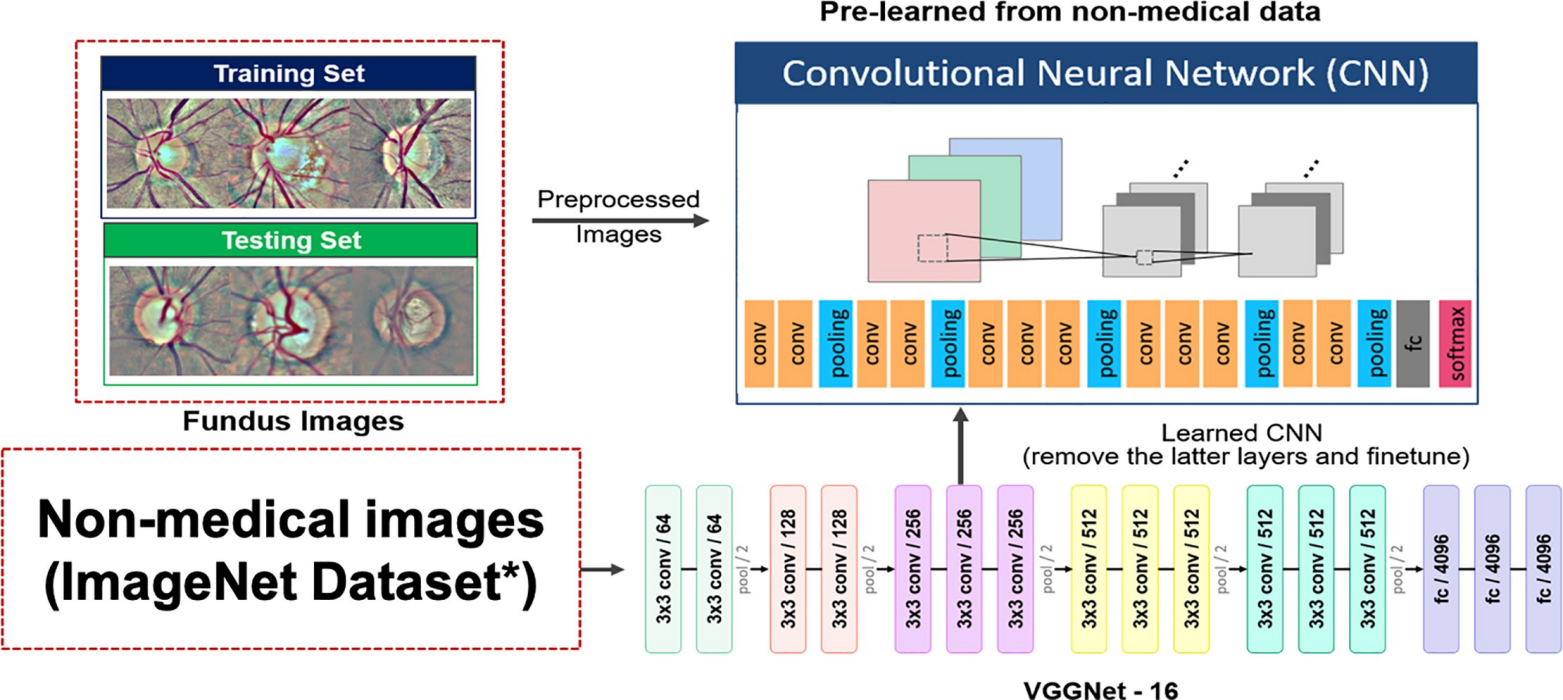
Transfer learning and VGGNet architecture. Under transfer learning strategy, the CNN model was initialized with the trained weight of VGGNet with a 3 x 3-sized filter. The final model contained convolutional layers, pooling layers, and the fc layer. VGGNet, invented by Visual Geometry Group from University of Oxford; CNN, convolutional neural network; conv, convolutional; fc, fully connected. *Russakovsky O, et al. ImageNet large scale visual recognition challenge. *IJCV*. 115, 211–252 (2015).

In our CNN model, preprocessed and qualified images were submitted for processing through a series of convolutional, nonlinear functions (ReLU), pooling, fully connected (FC) layers, and finally softmax loss layer, which incorporated the concept of cross entropy and softmax layer to provide a probability value for the final classification of each image. Moreover, Stochastic Gradient Descent was performed for optimization to minimize the loss of function. Instead of training a model from scratch, transfer learning was performed using VGGNet (invented by Visual Geometry Group from University of Oxford) [23], a pre-trained CNN model through the following steps: removal of top layers such as the FC layers; retraining the CNN model initialized from the trained weights of VGGNet with a series of convolutional layers of 3 x 3 filter size (Fig 3) [24,25]. Regularization methods, such as data augmentation and dropout layer, were used to improve the condition of convergence and prevent overfitting.

### Cross-validation of the CNN model

We performed 5-fold cross validation to evaluate the performance and to avoid overfitting of the CNN model. Within each fold, the combined images were partitioned into independent training and validation sets using an 80–20 percentage split. The performance of each fold was evaluated not only on the validation sets, but also the test images. The parameters of the fold with best performance were selected as the final model, unless there were significant variation on the performance of each fold.

### Class activation map (CAM) for visualization

**Fig 4:**
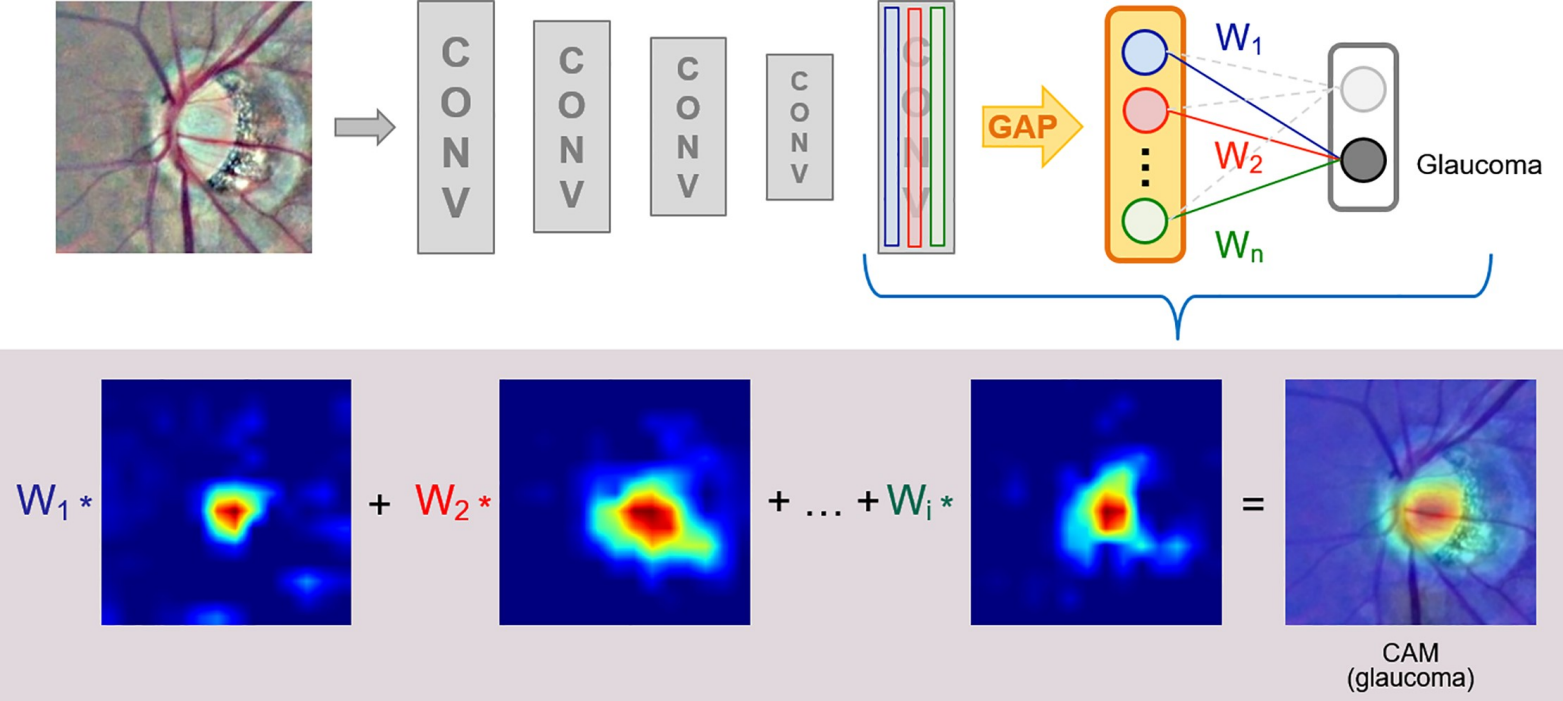
Class activation map attached to the final layer of the proposed CNN model. CAM revealed the important areas for the final classification and was generated using GAP with the multiplication of each channel by the given weight from the fully connected (fc) layer (W1, W2…Wn). CAM, class activation map; CNN, convolutional neural network; CONV, convolutional; GAP, global average pooling.

CAM was applied to identify features recognized by the proposed CNN model to differentiate between the glaucomatous and normal discs [26]. Through global average pooling (GAP) performed on the convolutional feature maps and an FC layer that produced the desired output, the important regions of each image were identified by projecting the weights of the output layer back on to the convolutional feature maps (Fig 4) [27].

### SVM classifier based on enlarged CDR

**Fig 5:**
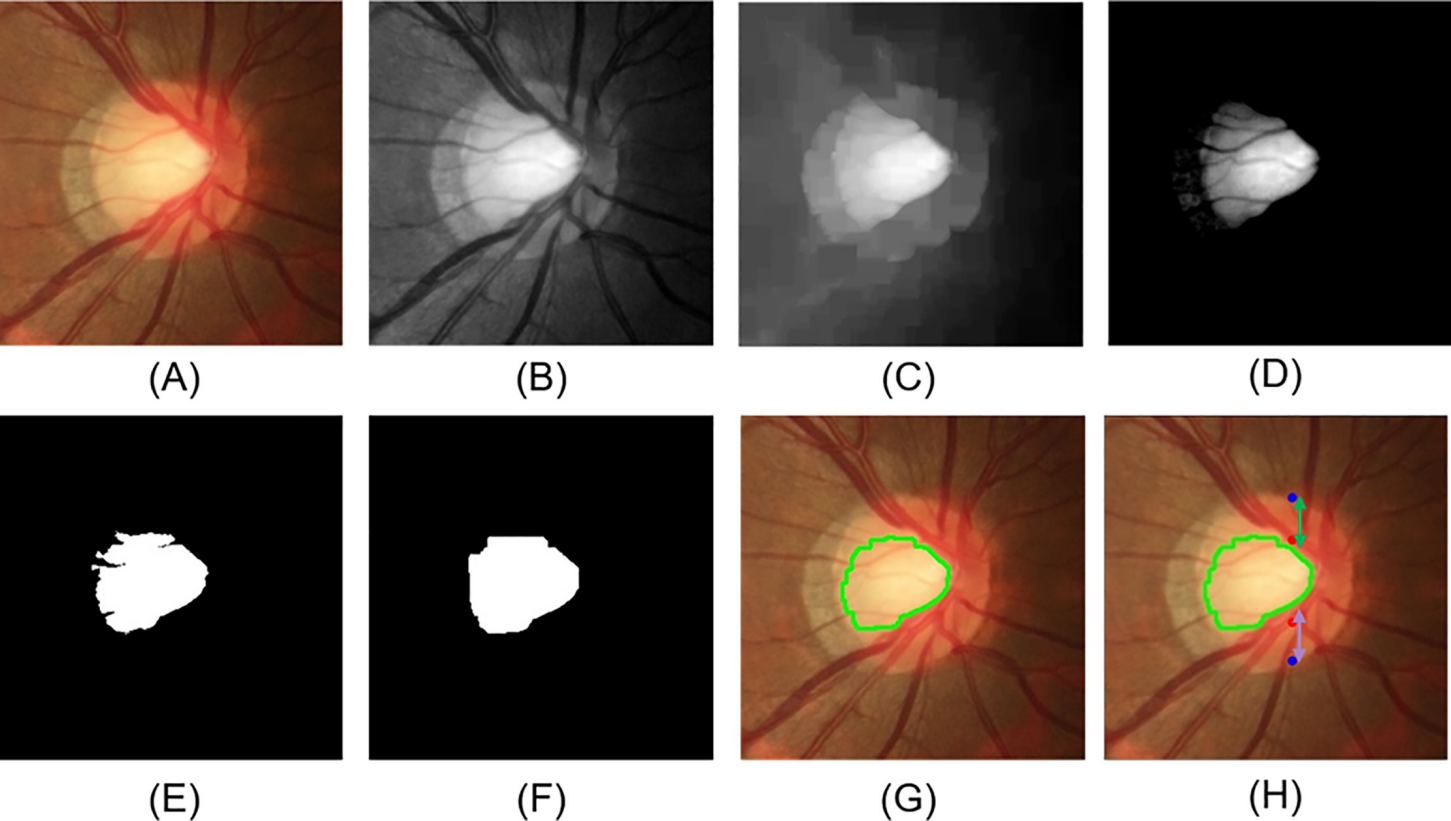
Process to determine the disc margin, cup area, vertical cup-to-disc ratio, and superior and inferior neuroretinal rim-to-disc ratio. (A) Color fundus image after ROI extraction; (B) green and red channel processing; (C) removal of the vessels; (D) processing by subtracting the mean and standard deviation; (E) K-means clustering; (F) morphological operations to remove noise; (G) the cup boundary (green line); (H) vertical disc diameter (between blue points), cup diameter (between red points), superior rim (green arrow), and inferior rim (purple arrow). ROI, region of interest.

The characteristic changes of the glaucomatous disc, including an increased value of CDR, and thinning of the superior or inferior neuroretinal rim were selected as the features for SVM classifier. Preprocessed images were first processed with the green and red channel. The disc margin was determined by the red channel, and the cup area by green channel. The vertical disc diameter was decided according to the superior and inferior junctional points of the disc boundary along the vertical meridian. K-means clustering was used to determine the cup area after removing the redundant information by subtracting the mean and standard deviation from the green channels, and the K value was set to 2 as dividing the preprocessing image into two regions (Fig 5) [28]. The CDR was calculated as the vertical cup length divided by the vertical disc diameter; the rim-to-disc ratio was calculated as the remaining part at each side including the superior and inferior neuroretinal rim width divided by the vertical disc diameter. The three features were input into the SVM classifier with RBF kernel for classification. Prediction was implemented by using a grid search and the SVM model provided by scikit-learn [29].

### Statistical analysis

The performance of our model was evaluated by the indices of sensitivity, specificity, and accuracy defined as the sum of correct classification of both the healthy and glaucomatous images divided by the total number of test images. The receiver operating characteristic (ROC) curve and area under the ROC curve (AUC) were plotted using the python scikit-learn library according to the definition, wherein, the true positive rate is the Y-axis, and the false positive rate is the X-axis. Nonparametric tests were used to compare the clinical characteristics between images with correct and incorrect prediction because most measurements of the test images were not normally distributed. Mann-Whitney U test was used for continuous variables, and chi-square test for categorical variables. The analyses were conducted using SPSS (version 18.0.0, SPSS Inc., Chicago, IL, USA).

## Result

### Diagnostic performance of the CNN models

**Fig 6:**
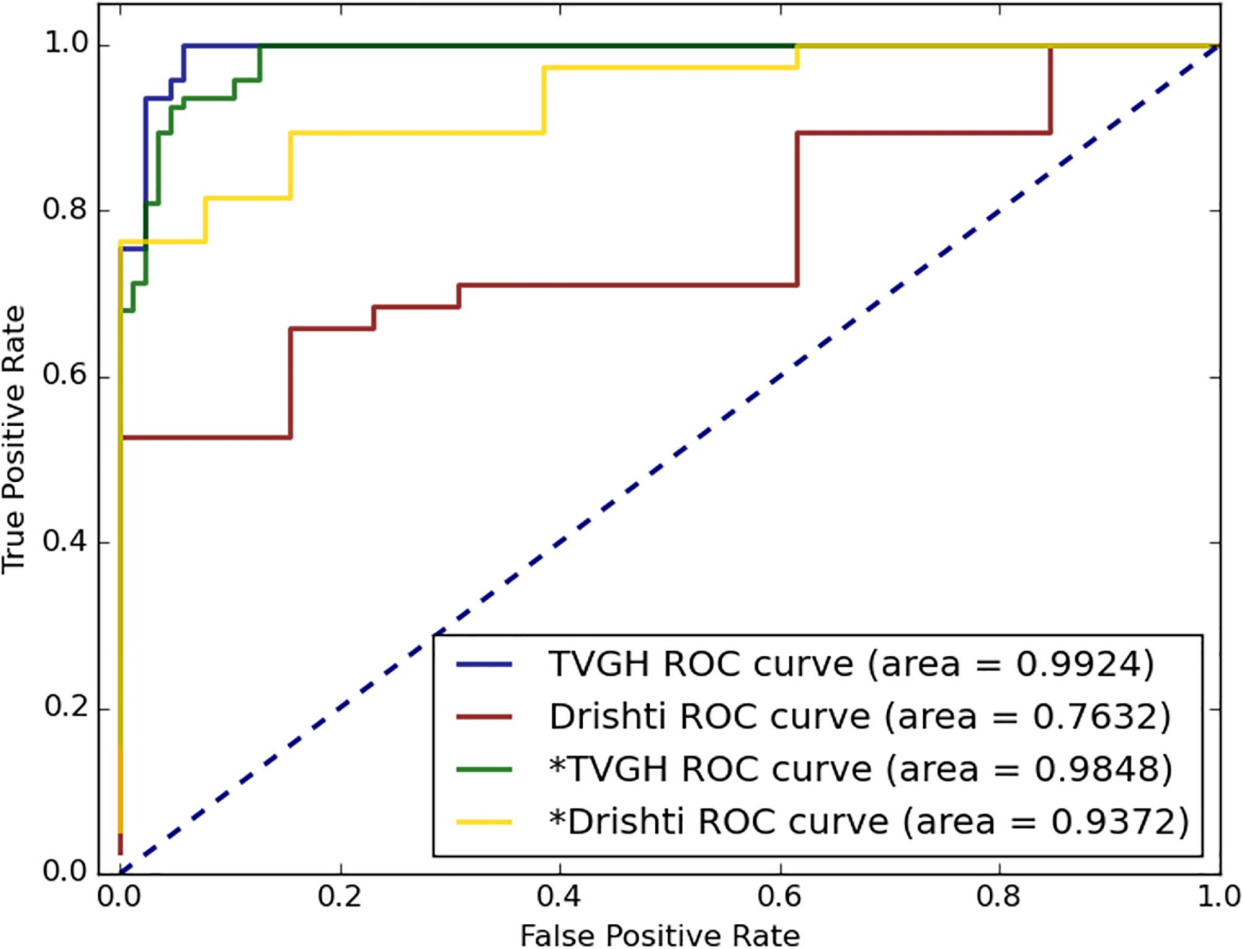
ROC curve of the CNN models to differentiate between the normal disc and glaucomatous disc. The TVGH ROC curve indicated the result through the TVGH-CNN model established based on the TVGH training images to predict the TVGH test images. The Drishti ROC curve indicated the result through the CNN model established using the Drishti-GS training images to predict the Drishti-GS test images. *TVGH and *Drishti used the combined TVGH and Drishti training dataset to predict the TVGH and Drishti test images, respectively. ROC, receiver operating characteristic; CNN: convolutional neural network.

In a total of 187 test images from the Taipei Veterans General Hospital (TVGH) dataset, the diagnostic accuracy of the TVGH-CNN model was 95.0%, sensitivity was 95.7%, and specificity was 94.2% in differentiating the glaucomatous eyes from the normal eyes; the AUC of the TVGH-CNN model for identifying glaucomatous eyes was 0.992 (Table 2 and Fig 6). However, the accuracy of the TVGH-CNN model used to classify the 51 test images of the Drishti-GS dataset was comparatively lower at 33.3%, which indicated poor generalizability of the TVGH-CNN model. Two approaches were used to overcome this problem: the first was to fine-tune the TVGH-CNN model using the training images of the Drishti-GS dataset; the second was to develop an ensemble model which used CDR for classification when the confidence score of the CNN classifier was low.

**Table 2:** Diagnostic capability of the CNN model, SVM model, ensemble model and fine-tuned CNN model in identifying glaucomatous disc.

	Dataset of test images							
	TVGH				Drishti-GS			
**Models**	**Accuracy**	**Specificity**	**Sensitivity**	**AUC (95% CI)**	**Accuracy**	**Specificity**	**Sensitivity**	**AUC (95% CI)**
**TVGH-CNN model**^*a*^	95.0%	94.2%	95.7%	0.992 (0.984–1.0)	33.3%	100%	10.0%	0.763 (0.632–0.894)
**SVM model**^*a*^	76.7%	78.1%	75.5%	0.806 (0.740–0.872)	72.5%	76.9%	71.0%	0.783 (0.617–0.950)
**Ensemble model**	92.8%	95.4%	90.4%		80.3%	92.3%	76.3%	
**Fine-tuned T&D-CNN model**^***b***^	93.9%	95.4%	92.6%	0.984 (0.972–0.998)	80.3%	100%	73.6%	0.937 (0.874–1.0)

^*a*^The model was constructed based on the TVGH training dataset.

^b^The model was constructed based on the mixed training images of the TVGH and Drishti-GS datasets (T&D-CNN model). CNN, convolutional neural network; SVM, support vector machine; TVGH, Taipei Veterans General Hospital; AUC, area under the curve; CI, confidence interval.

**Fig 7:**
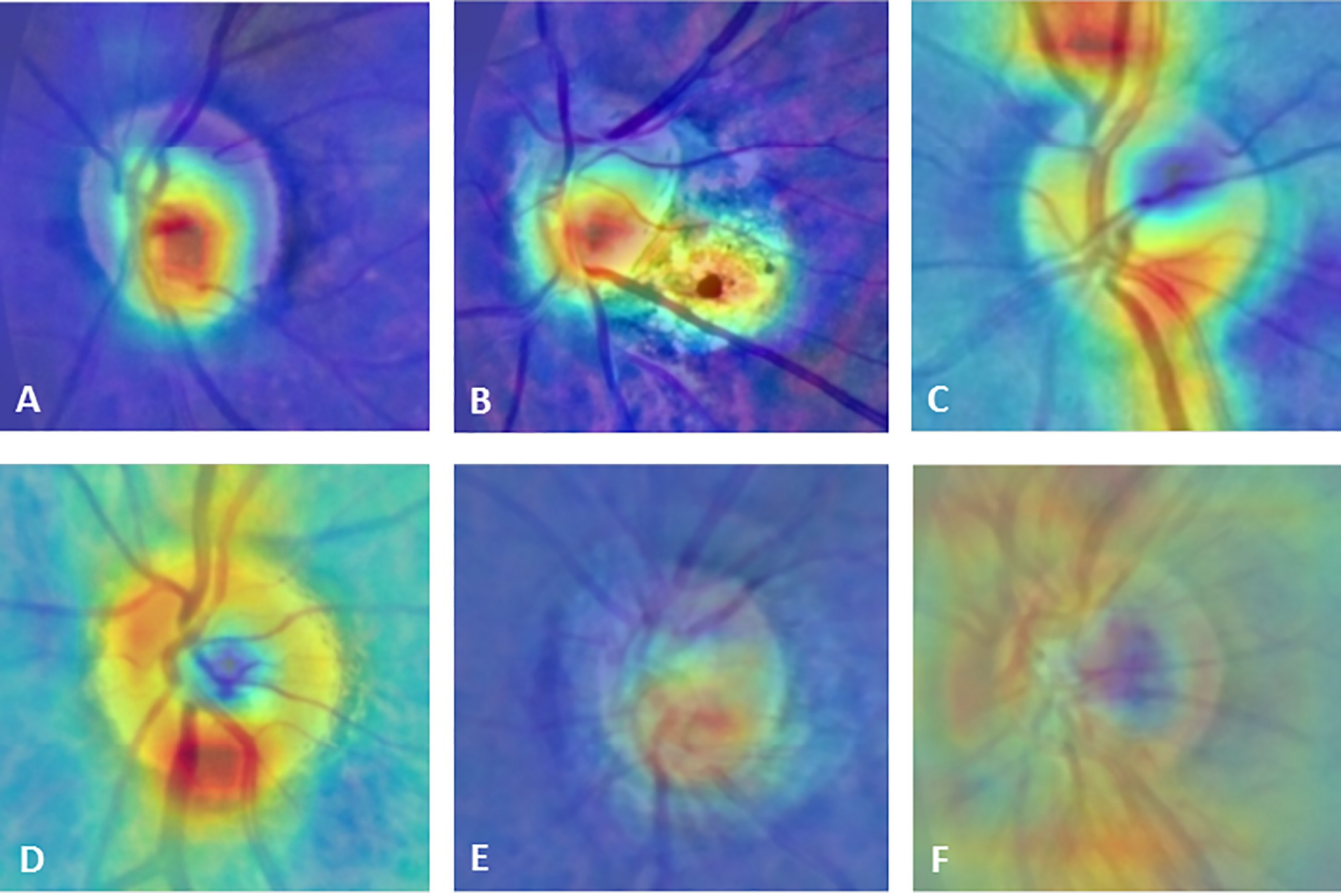
Representative CAMs and superimposed images showing different patterns between the glaucomatous and healthy eyes. The hot spots (red color) were localized at the cup and the region of parapapillary atrophy in the glaucomatous eyes (A, B), and at the neuroretinal rim or along the nerve fibers bundles in the healthy eyes (C, D). The superimposed images with CAM centered at the disc, showed the different patterns of CAM between the glaucomatous (E) and healthy eyes (F). CAM, class activation maps.

Fifty training images of the Drishti-GS dataset were added to the TVGH training dataset to establish a fine-tuned CNN model (T&D-CNN model), which improved the classification accuracy of the CNN model applied to the Drishti-GS test images. The diagnostic accuracy of the T&D-CNN model for Drishti-GS test images was improved to 80.3%, with a sensitivity of 73.6%, specificity of 100%, and an AUC of 0.937 (Table 2 and Fig 7). To determine optimum incorporation of the limited training images of Drishti-GS dataset into the TVGH dataset, we analyzed the effects of preprocessing, augmentation, and ROI extraction of the training images on the performance of the T&D-CNN model. The result indicated that ROI selection increased the performance of the model. For dataset with limited images such as Drishti-GS, preprocessing and augmentation may increase the model performance (Table 3).

**Table 3:** Impact of image processing on the performance of the proposed CNN model in identifying glaucomatous discs using a mixed training dataset including training images of Drishti-GS and TVGH datasets.

	Dataset of test images					
	TVGH			Drishti-GS		
Methods included in image processing	Specificity	Sensitivity	AUC (95% CI)	Specificity	Sensitivity	AUC (95% CI)
**All** ^*a*^	95.4%	92.6%	0.984 (0.972–0.998)	100%	73.6%	0.937 (0.874–1.0)
**All except ROI selection**	93.4%	89.4%	0.978 (0.960–0.996)	100%	55.2%	0.887 (0.789–0.984)
**All except preprocessing**	97.7%	94.6%	0.993 (0.985–1.0)	84.6%	84.2%	0.897 (0.794–0.999)
**All except augmentation**	95.4%	96.8%	0.990 (0.997–1.0)	92.3%	68.4%	0.925 (0.849–1.0)

^*a*^ROI selection, preprocessing, and augmentation were performed. The augmentation was performed by rotation, shifting, sheering, and zooming with a final image number of 30000 using the 50 training images from the Drishti-GS dataset. CNN: convolutional neural network; TVGH, Taipei Veterans General Hospital; AUC, area under the curve; CI, confidence interval; ROI, region of interest.

### Diagnostic performance of the ensemble model

An ensemble model for classification was also established with the aim to avoid missing subjects with moderate to advanced disease, which used a SVM classifier based on CDR for final classification when the probability value of the CNN classifier was lower than the threshold of 0.85. The probability value refers to the binary classification confidence of the CNN classifier for the predicted classification. The threshold value was selected by calculating the sensitivity and specificity of the ensemble model with different threshold values starting from 0.5 with an increment of 0.05 till 1.0. The ensemble model had highest sensitivity and specificity on test images including TVGH and Drishti-GS datasets at a threshold value of 0.85. The SVM classifier, which was established based on the TVGH dataset, had a diagnostic accuracy of 76.7% and 72.5% for the TVGH and Drishti-GS test images, respectively. The ensemble model had better diagnostic accuracy for the Drishti-GS test images than that of the TVGH-CNN model, but similar diagnostic accuracy as that of the fine-tuned T&D-CNN model; moreover, there was no increase in the overall diagnostic accuracy for the TVGH test images than that of the TVGH-CNN model (Table 2).

### Factors affecting the performance of the CNN classifier

To understand the potential factors leading to misclassification through our model, we compared the clinical characteristics for the images with correct and incorrect prediction (Table 4). For glaucoma, the images being correctly classified as glaucoma had larger CDR and worse visual field defect than those being incorrectly classified. All images with GON that were incorrectly classified as normal had a mean deviation of better than -6 dB. The images of healthy eyes that were incorrectly classified as glaucoma had larger CDR values than those that were correctly classified.

**Table 4:** Clinical characteristics for the test images and relationship with the prediction of the deep learning algorithm.

	POAG			Control		
	CNN prediction			CNN prediction		
	Correct N = 87	Incorrect N = 7	P- value^a^	Correct N = 83	Incorrect N = 4	P- value^a^
**Age (years)**	58.85±15.71	62.00±2.52	0.389	62.26±19.55	51.75±23.26	0.297
**Sex (Female, %)**	33 (37.9%)	1 (14.28%)	0.210	49 (59.03%)	4 (100%)	0.101
**CDR**	0.80±0.11	0.67±0.21	0.037	0.41±0.14	0.57±0.10	0.018
**Visual field**						
**Mean deviation (dB)**	-6.49±5.47	-2.16±2.27	0.015			
**PSD (dB)**	6.28±3.72	2.97±1.51	0.011			
**Average RNFL thickness (μm)**	72.79±11.06	80.71±10.92	0.750			

Seven images with wrong automated ROI selection were excluded in the analysis.

Values are presented as mean ± SD or percentage (%).

^*a*^ Mann-Whitney U test was used for continuous variables, and Chi-square test for categorical variables. POAG, primary open angle glaucoma; CNN, convolutional neural network; D, diopter; CDR, cup-to-disc ratio; PSD, pattern standard deviation; RNFL, retinal nerve fiber layer.

### Visualization of predictive features

CAM was used to reveal features recognized by our CNN model for classification. Areas critical for the classification were marked as red, while areas with low impact as blue. The CAM was different between the glaucomatous and healthy eyes. The hot spots were located most commonly at the cup area and region of parapapillary atrophy in the images of the glaucomatous eyes, but at the neuroretinal rim or along the retinal nerve bundles in the images of the healthy eyes. The superimposed images revealed the difference between the two groups (Fig 7).

## Discussion

This study found that CNN-based automated glaucoma detection using color fundus images acquired by a desktop fundus camera achieved high accuracy on test images whose quality is similar to that of the training dataset. However, the generalizability of the classification model depended on the diversity of the training data. Addition of a limited number of images from the target test population to the training dataset significantly increased the accuracy of the CNN detection model. With this modification, CNN achieved similar or better diagnostic performance than the ensemble model that considered CDR. CAM revealed that our CNN model captured characteristics areas of the optic disc as that through human perception to identify glaucomatous changes.

The key to achieving accurate classification of retinal images using deep learning is the number and quality of the training images [19,20]. Unlike the diagnosis and grading of diabetic retinopathy which uses only fundus photographs, the diagnosis of glaucoma involves various structural and functional examinations [18]. It is very difficult to collect tens of thousands of glaucomatous fundus images with definite diagnosis and complete profiles of functional and structural measurements. This issue was settled in some studies aiming for building CNN classifier using large number of fundus photographs by identifying referable GON but not definite GON based on findings of the fundus images and agreement between doctors [16,30]. The major limitation of this approach is that one cannot evaluate the diagnostic performance of any proposed system for images with different level of disease severity. Christopher et al reported that the performance of deep learning algorithm differed according to the severity of GON [15]. In line with their findings, glaucomatous eyes at an earlier stage were more likely to be misclassified as normal in our model. Thus, diagnostic performance of deep learning algorithms as currently available diagnostic tool for glaucoma may vary according to the disease severity and is more likely to miss early GON [15].

The generalizability of our deep learning model trained with limited fundus images was inadequate. Lack of diversity of our database may explain the poor generalizability of our model; however, it is unclear if the generalizability of a deep learning model would be improved by using a larger number of images of various origins because verification of the CNN model on other database has not been thoroughly evaluated [15,16]. Phene et al reported that a deep learning algorithm trained with 58033 fundus images still had variable diagnostic performance in detecting GON on two validation datasets with an AUC of 0.940 and 0.858 [17]. Liu et al used 241032 images from 68013 patients to build a CNN classifier for automated glaucoma detection [30]. However, even with the largest image dataset to date, the CNN classifier still had suboptimal performance on images from subjects of different ethnicities and of variable qualities, with an AUC dropping from 0.996 with internal validation set to 0.923 and 0.823, respectively. Gulshan et al. found that the performance of a deep learning model in detecting diabetic retinopathy reached a plateau at around 60000 images [19]. Therefore, to improve the generalizability of a CNN classifier for glaucoma detection, approaches other than expanding the number of images of the training dataset should also be considered.

We proposed two approaches to improve the generalizability of our model: The first was to fine-tune the CNN model with limited number of images from the target test dataset which could dramatically improve the diagnostic performance of this mixed model for images of different origin. This approach is not novel but practical, with an aim to build clinic-based glaucoma screening algorithm using limited images from each clinic when neither a universal deep learning algorithm for glaucoma detection nor the access to large-scale glaucoma image datasets is available. Similar finding was noted in Diaz-Pinto et al’s study [31]. They used 5 public datasets to evaluate the generalizability of their CNN model and found that the diagnostic performance of the model varied according to whether the training images of the test dataset were included for model training or not with an AUC varied from 0.9605 to 0.7678, respectively. The second was to use an ensemble model that considered the CDR when the confident score of the CNN classifier was low. The original goal of the ensemble model was to avoid missing cases with advanced GON. However, the results revealed that the fine-tuned CNN model achieved similar diagnostic performance as that of the ensemble model. Therefore, when a large image dataset of images with definite diagnosis of GON is not available, a CNN model could be first constructed using hundreds of images with high quality and then fine-tuned using some images from the target test population to improve the diagnostic power of the model in the target test population.

The CAM revealed that the class-discriminative regions of the CNN model co-localized with the known characteristics of GON. The hot spots of the CAM were located at the cup area in most images that were correctly classified as glaucoma, but at the neuroretinal rim and nerve bundles in the images of healthy eyes. This finding agreed with the report of Christopher et al that the inferior and superior regions of the neuroretinal rim are the most important parts for distinguishing between the eyes with GON and healthy eyes through deep learning algorithm [15]. These findings clarified the classification of glaucoma based on CNN model and may enable further modification of the CNN model to achieve higher accuracy.

Our study has some limitations. The major limitation is that the training dataset was collected at one medical center with limited number of images, which may lead to questionable generalizability of our model. As expected, our model showed poor performance on test images of other origin, which could be overcome by using the fine-tuned model. Further evaluation to verify the generalizability of deep learning algorithm for glaucoma detection is needed since the fundus images taken for glaucoma diagnosis may vary in layout, clarity, and visibility of the RNFL, especially for population-based glaucoma screening. The second limitation of our study is that ROI centered at the ONH was used to construct the model to reduce the image size and complexity, which may result in loss of information and complicated preprocessing at clinical application. However, ROI extraction increased both the sensitivity and specificity of the model as compared with those of the whole images (Table 3). Third, we did not verify the performance of our approaches using different deep learning algorithms in addition to VGGNet because the performance and generalizability of the TVGH-CNN model did not change significantly with other CNN algorithms, such as AlexNet, GoogLeNet, or Xception (data not shown). In line with our findings, Christopher et al’s study revealed that the difference of VGGNet, Inception, and ResNet50 in detecting GON is marginal, with an AUC of 0.89, 0.91, and 0.91 respectively [15]. Finally, cases with anomalous or extreme disc appearance and retinopathy except for drusen were excluded from the dataset; hence, our model may not be applicable to such cases. Meanwhile, we did not measure disc size, not count disc hemorrhage or peripapillary atrophy in this study, therefore the impact of disc size, disc hemorrhage and peripapillary atrophy on the performance of CNN classifier is unknown.

## Conclusions

The proposed deep learning model trained with limited fundus images achieved high accuracy in identifying GON on images of similar quality, and did not misclassify any cases with moderate to advanced disease. The model could be successfully fine-tuned to increase its diagnostic accuracy for images of different origins, which might be a feasible approach to build CNN classifier for clinic-based glaucoma screening. The CAM revealed that the model captured similar characteristics in identifying GON as those through human perception. Based on these findings, deep learning assisted detection of GON may be a viable option to assist glaucoma screening, but the generalizability of a deep learning model must be verified first before being applied to the targeted test population.
